# Empirical transition probability modeling of continuous glucose monitoring data

**DOI:** 10.1371/journal.pone.0358361

**Published:** 2026-10-06

**Authors:** Yuanzhi Yu, Margaret Banker, Jennifer Sherr, Denise M. Scholtens

**Affiliations:** 1 Northwestern University Feinberg School of Medicine, Department of Preventive Medicine, Division of Biostatistics and Informatics, Chicago, Illinois, United States of America; 2 Department of Pediatrics, Yale School of Medicine, New Haven, Connecticut, United States of America; Kwara State University, NIGERIA

## Abstract

**Introduction:**

Continuous glucose monitoring (CGM) produces rich time series data. Standard metrics such as time-in-range summarize glycemia but miss the sequence of changes. We aimed to characterize glucose dynamics using empirical transition probability techniques.

**Materials and Methods:**

Using publicly available data for 244 participants who enrolled in the Insulin-Only Bionic Pancreas (IOBP2) randomized clinical trial, we mapped each CGM reading to one of five states: < 54, 54–69, 70–180, 181–250, or >250 mg/dL. We counted state changes between consecutive readings collected every 5 minutes. For each participant, we built a 5 × 5 transition probability matrix and averaged matrices within comparator groups. Statistical inference using estimated transition probability matrices was compared to inferences based on standard metrics including mean glucose, standard deviation, coefficient of variation, time-in-range metrics, Glycemia Risk Index (GRI), Mean of Daily Differences (MODD), and Mean Amplitude of Glycemic Excursions (MAGE).

**Results:**

Transition probability analysis showed more frequent moves from hyperglycemia (181–250 or >250 mg/dL) to the in-range (70–180 mg/dL) state for the bionic pancreas that was evaluated in the IOBP2 trial compared to control. This pattern appeared in both adults and in children and persisted through the trial. Conventional CGM metrics also showed treatment differences in mean glucose, GRI, and time in range; MODD, MAGE, and coefficient of variation showed less consistent differences between treatment groups.

**Conclusion:**

Transition probability matrices provide an informative summary of glucose dynamics that complements established CGM metrics. In IOBP2, this approach highlighted improved patterns of recovery from hyperglycemia with the bionic pancreas compared with control. Future studies should determine whether transition-based summaries are associated with clinically meaningful outcomes.

## Introduction

The advent of wearable continuous glucose monitoring (CGM) technologies revolutionized diabetes management both in clinical practice and research settings by providing detailed real-time glucose readings on the order of minutes [1–5]. Unlike traditional capillary blood glucose measurements, commonly called “fingersticks”, which might miss critical fluctuations, CGM generates dense, time-ordered data points over the period of wear. The dynamic view provided by CGM also offers improvement over hemoglobin A1c (HbA1c) for understanding glycemia. While the Diabetes Control and Complications Trial demonstrated risk of long-term complications associated with HbA1c that motivates its frequent use as the primary endpoint for clinical trials [6], HbA1c is impacted by red blood cell lifespan, glycation rate of an individual, and certain disease states (hemoglobinopathy and end stage renal disease) [7]. HbA1c is valuable for summarizing long-term glycemia, but it cannot capture the short-term fluctuations and rapid glucose changes that CGM data detect [8–10].

Despite their longitudinal richness, CGM data are often collapsed into simple summary statistics such as time-in-range (TIR), defined as the percentage of time spent between 70–180 mg/dL. Although adoption of TIR as a surrogate for long-term complications has historically been cautious, recent studies provide stronger evidence that CGM-derived measures, including TIR and related metrics, can stratify risk [11,12]. Further, these studies support the goal of achieving >70% TIR [1]. Yet, TIR only describes the proportion of time within a specific range and cannot distinguish whether glucose changes are gradual or involve sudden spikes and drops [8,13]. This concern mirrors the long-standing criticism of HbA1c: it is an average. HbA1c may fail to reflect true glycemic status because it summarizes only average tendency. By analogy, bucketing CGM into TIR truly may not capture the whole picture. For example, two individuals can both have TIR = 75%, yet one may have frequent excursions while the other remains relatively stable.

To address these limitations, several metrics exist to capture different types of glycemic variability from CGM data. The Glycemia Risk Index (GRI) combines weighted contributions of clinically important hypo- and hyperglycemia into a single summary score [14]. Mean Amplitude of Glycemic Excursions (MAGE) measures the average size of significant glucose swings within a day, focusing on the most prominent peaks and valleys [15]. Mean of Daily Differences (MODD) looks at how much glucose readings differ from one day to the next, giving a sense of day-to-day stability [16]. The coefficient of variation (CV) quantifies glucose variability as a percentage of the mean, with values greater than 36% generally indicating instability of glucose levels [1]. Despite their value, each metric has drawbacks [8,13,17–20]. For instance, MAGE ignores smaller and potentially important changes, and MODD does not capture within-day variability or the precise sequence of glucose changes.

Advanced analytic methods increasingly use dynamic features of CGM time series to describe glycemic patterns. Montaser et al. encoded CGM readings into eight glycemic states and used the resulting transition probability matrices to calculate an entropy-rate marker that distinguished participants with and without diabetes and differentiated type 1 from type 2 diabetes [21]. Other work has examined how long CGM must be worn to obtain stable estimates of time-in-range and time-below-range measures [22]. Hidden Markov and hidden semi-Markov models have also been applied to CGM data for calibration, forecasting, and latent-state inference [23–25]. Together, these studies show that sequential methods can describe state persistence, movement between states, and latent glucose patterns that are not represented by a single summary value. To provide a simple and intuitive summary of transitions between clinically defined states such as normoglycemia, hypoglycemia, and hyperglycemia, we estimate empirical transition probability matrices that quantify the probability of moving between predefined glucose states across consecutive time intervals. This approach preserves temporal ordering and provides information that complements static summaries. We applied this method to CGM data from the Insulin-Only Bionic Pancreas (IOBP2) trial, which evaluated the efficacy of the insulin-only iLet Bionic Pancreas (BP) system for improving HbA1c in individuals with type 1 diabetes.

## Materials and methods

### Study design and population

This analysis utilized publicly available data from the IOBP2 trial (ClinicalTrials.gov NCT04200313), obtained from the JAEB Study Website (https://public.jaeb.org/datasets/diabetes, downloaded October 20th 2025). IOBP2 was a multicenter, randomized controlled trial that evaluated the efficacy and safety of the BP system compared to standard care (Control) in participants with type 1 diabetes. IOBP2 enrolled 440 participants aged 6–79 years, who were diagnosed with type 1 diabetes at least one year prior to enrollment. Participants were eligible if they had been on a stable insulin regimen (insulin pump or multiple daily injections) for at least three months prior to enrollment [26]. Participants were randomized into treatment arms separately based on age:

- **Child participants (ages 6–17):** randomized 2:1 into either the BP or Control arm.
- **Adult participants (ages 18–79):** randomized 2:2:1 into the BP, iLet Bionic Pancreas using ultra-rapid insulin (Fiasp), or Control arm. Participants who received Fiasp were excluded from this analysis.

Throughout the trial, CGM data were collected with Dexcom G6 sensors which collect glucose measurements every five minutes.

The IOBP2 trial was designed as a 13-week intervention. For this analysis, we examined CGM data according to five periods:

- **Baseline:** All CGM data collected prior to each participant’s randomization date.
- **Weeks 1–12 (RCT Overall):** The 12 weeks following randomization, using only weeks 1–12 due to substantial missing data in week 13.
- **Weeks 1–4 (RCT Sub-period 1):** The first 4 weeks after randomization.
- **Weeks 5–8 (RCT Sub-period 2):** Weeks 5–8 after randomization.
- **Weeks 9–12 (RCT Sub-period 3):** Weeks 9–12 after randomization.

For our analysis, we required that participants had at least 80% complete CGM data within each RCT Sub-period (weeks 1–4, 5–8, and 9–12). CGM data completeness for each participant and time period is depicted in Fig 1. For Baseline analyses, we required that participants had at least two weeks of CGM data prior to randomization. These data did not need to be sequential, following the same criteria as the primary trial [26].

**Fig 1:**
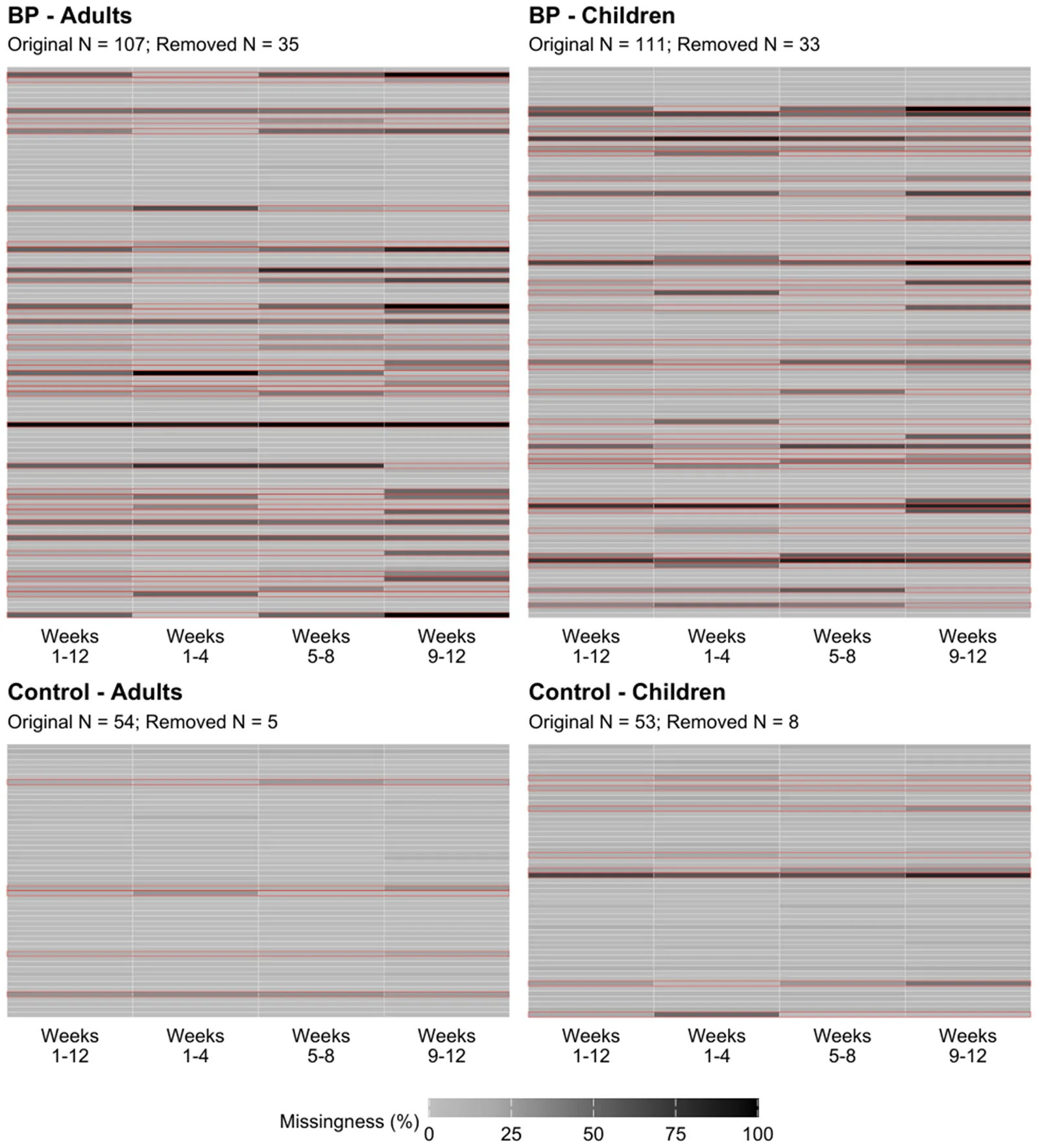
Each heatmap shows the percentage of missing CGM data for every participant across RCT Sub-periods (weeks 1-12, weeks 1–4, 5–8, and 9–12), stratified by treatment group (BP or Control) and age group (adults or children). Each row represents a participant; darker shades indicate a higher percentage of missing data. Red outlines mark participants who had less than 80% CGM completeness in at least one RCT Sub-period.

For period-specific group comparisons, we applied only the corresponding period’s coverage rule. In within-person analyses comparing RCT and Baseline periods, we applied both RCT completeness and Baseline completeness criteria to ensure each participant had sufficient data in both periods. The resulting analytic sample sizes, by age and treatment group, are summarized in S1 Table in S1 File ((Adults: RCT n = 121, Baseline n = 118, within-person Baseline+RCT n = 92; Children: RCT n = 123, Baseline n = 85, within-person Baseline+RCT n = 62)). Because hypoglycemia states were infrequent, some participants never entered these states in a given analytic subset. The number of such participants, by analysis type, treatment group, and age, is summarized in S2 Table in S1 File.

### Empirical transition probability estimation

Let $G_{t}$ denote the observed CGM glucose value at time t for a given participant, where t=1,2,…,T indexes chronologically ordered measurements for that individual. Each $G_{t}$ is mapped to a discrete glycemic state $X_{t}$ according to established clinical thresholds [1]:

1 Level 2 Hypoglycemia: $G_{t}<\text{54}$ mg/dL ($X_{t}=\text{1}$)
2 Level 1 Hypoglycemia: $\text{54}\leq G_{t}<\text{70}$ mg/dL ($X_{t}=\text{2}$)
3 In-range: $\text{70}\leq G_{t}\leq \text{180}$ mg/dL ($X_{t}=\text{3}$)
4 Level 1 Hyperglycemia: $\text{180}<G_{t}\leq \text{250}$ mg/dL ($X_{t}=\text{4}$)
5 Level 2 Hyperglycemia: $G_{t}>\text{250}$ mg/dL ($X_{t}=\text{5}$)

Here, S=5 is the total number of glycemic states, and $X_{t}\in \left\{\text{1},\text{2},\ldots ,\text{5}\right\}$.

For each participant, we constructed an empirical transition probability matrix of size S×S (here, 5×5), where the (i,j)th entry represents the probability of transitioning from state i at time t to state j at the subsequent eligible time $t^{\prime }$. To ensure transitions reflected actual glucose dynamics, we only considered consecutive CGM measurements $\left(X_{t},X_{t^{\prime }}\right)$ such that the time interval $\Delta t=t^{\prime }-t$ was greater than 4 minutes and did not exceed 6 minutes; transitions spanning longer and shorter intervals were excluded to minimize bias from missing data and device errors. Thus, each matrix entry summarizes the empirical probability of moving from state i to state j generally within a 5-minute window but at most for 4<Δt≤6 minutes.

Each row of the matrix represents all possible next-state transitions originating from a given state and sums to one:


$$\begin{array}{c} \sum_{j=\text{1}}^{S}P_{ij}=\text{1},\;\;\forall i\in \left\{\text{1},\text{2},\ldots ,S\right\}. \end{array}$$

The matrix for S=5 glycemic states has the form:

$𝐏=[\begin{array}{ccccc} P_{\text{11}} & P_{\text{12}} & P_{\text{13}} & P_{\text{14}} & P_{\text{15}}\\ P_{\text{21}} & P_{\text{22}} & P_{\text{23}} & P_{\text{24}} & P_{\text{25}}\\ P_{\text{31}} & P_{\text{32}} & P_{\text{33}} & P_{\text{34}} & P_{\text{35}}\\ P_{\text{41}} & P_{\text{42}} & P_{\text{43}} & P_{\text{44}} & P_{\text{45}}\\ P_{\text{51}} & P_{\text{52}} & P_{\text{53}} & P_{\text{54}} & P_{\text{55}} \end{array}]$

where $P_{ij}$ is the probability of moving from state i to state j in one eligible transition step, and i,j∈{1,…,5}. Note that j can equal i, so the matrix includes probabilities of remaining in the same state.

Let $n_{ij}^{\left(u\right)}$be the number of observed transitions from state i to state j for participant u (u=1,…,N), where N is the number of participants in the analysis group. Let $n_{i+}^{\left(u\right)}=\;\sum_{k=\text{1}}^{S}n_{ik}^{\left(u\right)}\;$be the total number of transitions observed from state i. When $n_{i+}^{\left(u\right)}>\text{0}$, the participant-specific empirical transition probability is


$$\begin{array}{c} P_{ij}^{\left(u\right)}=\frac{n_{ij}^{\left(u\right)}}{n_{i+}^{\left(u\right)}} \end{array}$$

For a given analysis group (defined by treatment arm, age group, and study period), we summarized transitions by averaging individual matrices row-wise among participants who entered the corresponding “from” state. Specifically, for each row i, let $N_{i}=\{u\in \text{group}:n_{i+}^{\left(u\right)}>\text{0}\}$. The group-level mean transition probability is


$$\begin{array}{c} P_{ij}^{\text{group}}=\frac{\text{1}}{N_{i}}\sum_{u\in N_{i}}P_{ij}^{\left(u\right)} \end{array}$$

If participant u never enters state i (so $n_{i+}^{\left(u\right)}=\text{0}$), the entire row i is undefined for that participant and is excluded only for that row; the participant still contributes to all other rows for states they visited. This row-wise rule is important for rare glycemic states, for example Level 2 hypoglycemia, because some IOBP2 participants never entered those states during a given analysis period.

For within-participant comparisons (e.g., RCT vs Baseline), we computed participant-level differences and then averaged them over participants with defined row i in both periods:


$$\begin{array}{c} \Delta P_{ij}^{\text{within}}=\frac{\text{1}}{N_{i}^{\text{within}}}\sum_{u\in N_{i}^{\text{within}}}\left(P_{ij,\text{RCT}}^{\left(u\right)}-P_{ij,\text{Baseline}}^{\left(u\right)}\right) \end{array}$$

where


$$\begin{array}{c} N_{i}^{\text{within}}=\left\{u\in \text{group}:n_{i+,\text{RCT}}^{\left(u\right)}>\text{0}\text{ and }n_{i+,\text{Baseline}}^{\left(u\right)}>\text{0}\right\}. \end{array}$$

This empirical approach provides interpretable summaries of glycemic state dynamics for both individual participants and groups. Rows based on rare from-states or small numbers of observed transitions should be interpreted cautiously because the corresponding probabilities may be less stable than rows based on common states such as in range or hyperglycemia. We therefore report the number of participants who never entered selected glycemic states in S2 Table in S1 File.

### Marginal probability of glycemic states

We also quantified the overall distribution of time spent in each glycemic state, regardless of sequence, by computing the marginal probability of occupying each state. The formal definitions are provided in the Appendix A in S1 File.

### Traditional glycemic metrics

In addition to computing empirical transition matrix probabilities, we also computed established glycemic metrics for the IOBP2 CGM data to capture both overall glycemic exposure and variability:

1 **Mean glucose** (mg/dL)
2 **Standard deviation** (SD, mg/dL)
3 **Coefficient of variation** (CV, %)
4 **Percent time within 70–180 mg/dL** (In range, %)
5 **Percent time below 70 mg/dL** (Level 1 Hypoglycemia & Level 2 Hypoglycemia, %)
6 **Percent time below 54 mg/dL** (Level 2 Hypoglycemia, %)
7 **Percent time above 180 mg/dL** (Level 1 Hyperglycemia & Level 2 Hyperglycemia, %)
8 **Percent time above 250 mg/dL** (Level 2 Hyperglycemia, %)
9 **Glycemia Risk Index** (GRI) [14]
10 **Mean Amplitude of Glycemic Excursions** (MAGE) [15]
11 **Mean of Daily Differences** (MODD) [16]

We provide the formal definitions of GRI, MAGE, and MODD in the Appendix A in S1 File.

### Statistical analysis

We estimated a participant-specific 5 × 5 empirical transition probability matrix for each study period. We then summarized group-level transition patterns by averaging individual matrices within strata defined by treatment arm, age group, and period. For within-person comparisons (RCT vs Baseline), we computed participant-level matrix differences and averaged these differences within each treatment arm and age group.

To quantify uncertainty in between-group differences in transition probabilities, we used participant-level bootstrap resampling within each treatment arm (1,000 replicates). In each replicate, we resampled participants with replacement, recomputed the group-level mean transition probability matrix for each arm, and then computed the between-arm difference for each of the 25 cells. We used the empirical bootstrap distribution of each cell-specific difference to form 95% confidence intervals. We also computed two-sided p-values for each cell based on this bootstrap distribution. To account for testing across 25 cells, we applied a Bonferroni correction, using a significance threshold of α=0.05/25.

We compared conventional CGM summary metrics between BP and Control using Wilcoxon rank-sum tests (p<0.05), noting that these unadjusted tests differ from the baseline-adjusted models used for the primary IOBP2 trial analyses.

## Results

### Glycemia across treatment and age groups

In the IOBP2 trial, primary analyses demonstrated that use of the BP led to significant improvements in HbA1c as compared to standard of care [26,27]. Our own re-analysis stratifies participants by age group (adults and children) and applies data completeness criteria (at least 14 days of CGM data before randomization, and 80% coverage in each RCT Sub-period) to the publicly available IOBP2 dataset, for which 92 adults and 62 children met the completeness criteria. We found similar trends in HbA1c as in the original trial publication: among adults, the BP group showed a reduction from HbA1c of 7.63% at randomization to 7.07% at week 13, while the Control group remained unchanged (7.46% to 7.46%). Pediatric participants also showed a decline in HbA1c with BP (from 7.71% to 7.37%), though the between-group difference at week 13 was not statistically significant (**Table 1**). These findings are consistent with the overall cohort results reported in the original trial [28].

**Table 1:** Mean (SD) glycated hemoglobin (HbA1c, %) at randomization and week 13 by treatment group and age. P-values are from Wilcoxon rank-sum tests comparing week 13 HbA1c between BP and Control within each age group. Data include only participants with at least 14 days of CGM data before randomization, and at least 80% CGM coverage in each RCT sub-period (weeks 1–4, 5–8, and 9–12).

Age Group	Time Point	BP	Control	p-value
**Adults (N = 92)** (BP 51, Control 41)	Randomization	7.63 (1.12)	7.46 (1.01)	
	Week 13	7.07 (0.61)	7.46 (0.89)	0.040
**Children (N = 62)** (BP 37, Control 25)	Randomization	7.71 (1.25)	7.82 (1.10)	
	Week 13	7.37 (0.78)	7.85 (1.05)	0.076

Table 2 summarizes mean CGM glucose by group and period during the IOBP2 trial. For adults, both groups started with similar mean glucose at baseline. The BP group showed a reduction from 183.7 mg/dL (SD 74.9) at baseline to 154.9 mg/dL (SD 54.9) during weeks 1–12. The Control group showed a smaller decline from 181.0 mg/dL (SD 64.5) to 171.1 mg/dL (SD 67.9). These results track closely with the patterns seen in HbA1c.

**Table 2:** Mean (SD) CGM glucose (mg/dL) by treatment, age group, and period.

Age Group	Baseline	RCT (Weeks 1-12)	Weeks 1–4	Weeks 5–8	Weeks 9–12
**Adults (N=92)**					
BP (N=51)	183.7 (74.9)	154.9 (54.9)	153.7 (53.4)	155.1 (55.0)	156.0 (55.3)
Control (N=41)	181.0 (64.5)	171.1 (67.9)	173.3 (69.8)	176.0 (70.4)	174.2 (69.4)
**Children (N=62)**					
BP (n=37)	175.3 (72.4)	169.9 (66.2)	169.7 (65.4)	170.6 (67.1)	171.7 (69.2)
Control (n=25)	196.8 (82.0)	187.1 (78.8)	182.8 (76.8)	185.9 (79.3)	185.7 (80.6)

In children, there was a notable imbalance in mean glucose at baseline for BP (n = 37) and Control (n = 25). In our analysis, children randomized to BP began the study with a lower average glucose of 175.3 (SD 72.4) mg/dL compared with 196.8 (SD 82.0) mg/dL in the Control group. This difference persisted throughout the RCT, with the BP group maintaining lower mean glucose during the intervention, while the Control group also showed reductions (weeks 1–12: BP 169.9 [SD 66.2] mg/dL vs. Control 187.1 [SD 78.8] mg/dL). Baseline glucose may affect the probability of remaining in range or moving between glycemic states and the baseline imbalance across treatment arms may therefore influence unadjusted between-group transition comparisons. In the original IOBP2 trial, primary and secondary outcome analyses were performed using linear models that adjusted for individual baseline values, thereby accounting for initial differences [26]. A separate report focused on youth found that the baseline mean glucose was similar between treatment arms when all enrolled youth participants were included [27]. While baseline adjustment is not currently incorporated into transition probability matrix estimation for across-group comparisons, the within-individual analyses do implicitly account for baseline glucose when comparing transition probabilities prior to and following treatment.

Fig 2 summarizes group-level marginal probabilities for each glycemic state, treatment, age group, and period. These represent the average proportion of time spent in each state. During the RCT period, BP resulted in higher overall time spent in range (70–180 mg/dL) and less time in hyperglycemic states, in both adults and children, compared to Controls. At baseline, children randomized to BP already had a greater proportion of TIR than those in the Control group, reflecting the imbalance in mean glucose noted above. These patterns closely reflect the original trial’s findings.

**Fig 2:**
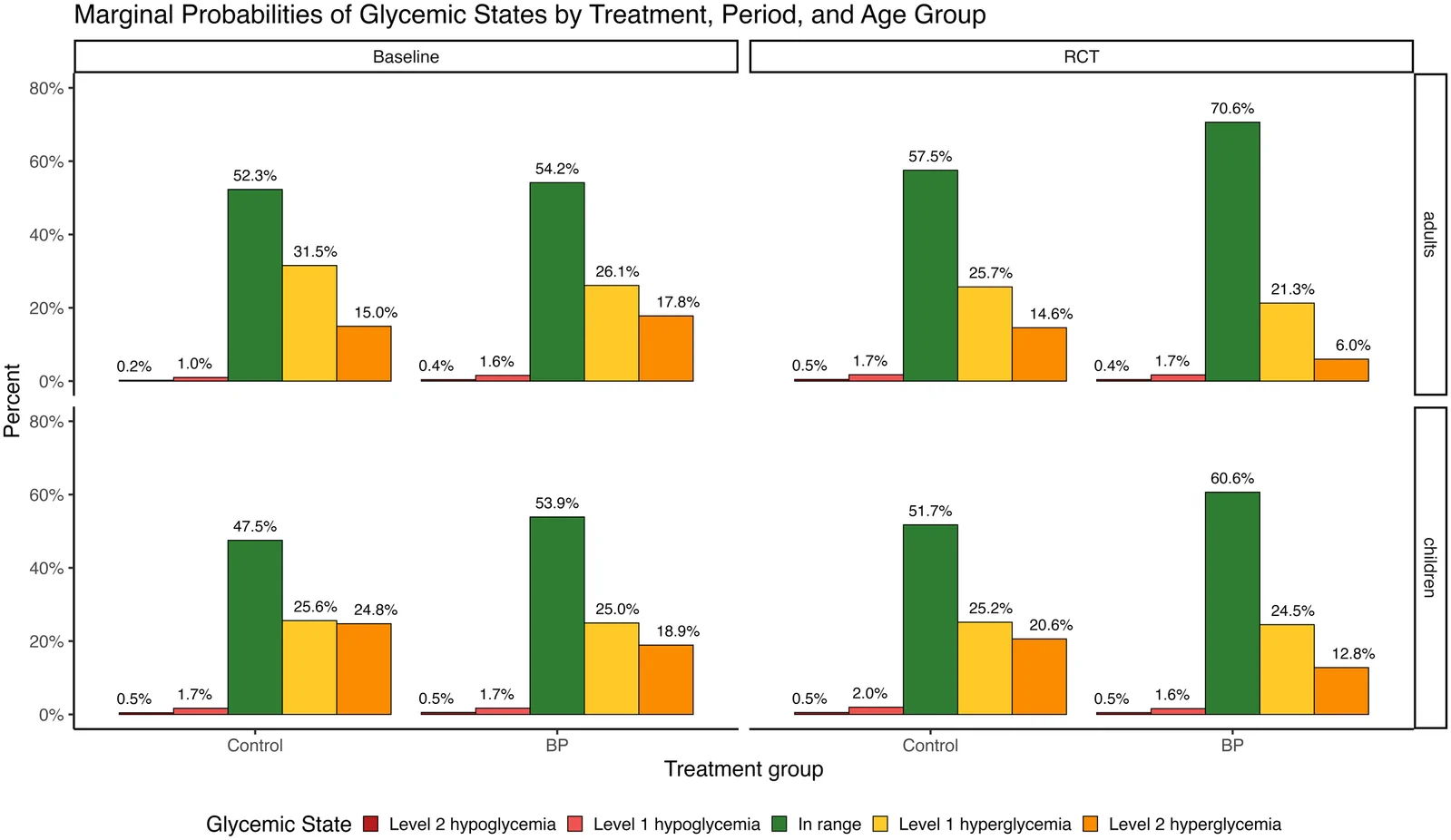
Bar plots display the mean percentage of time points in each glycemic state for adults and children in both BP and Control groups during Baseline and overall randomized trial (weeks 1–12) summaries.

### Empirical transition probability estimates

Fig 3 presents a grid of heatmaps that visualize the mean empirical transition probability matrices for each analysis group, stratified by age (adults, children), treatment arm (BP, Control), and study period (Baseline, RCT, Weeks 1–4, 5–8, 9–12). Each heatmap shows the within-group average probability of transitioning from one glycemic state to another in a specific time period.

**Fig 3:**
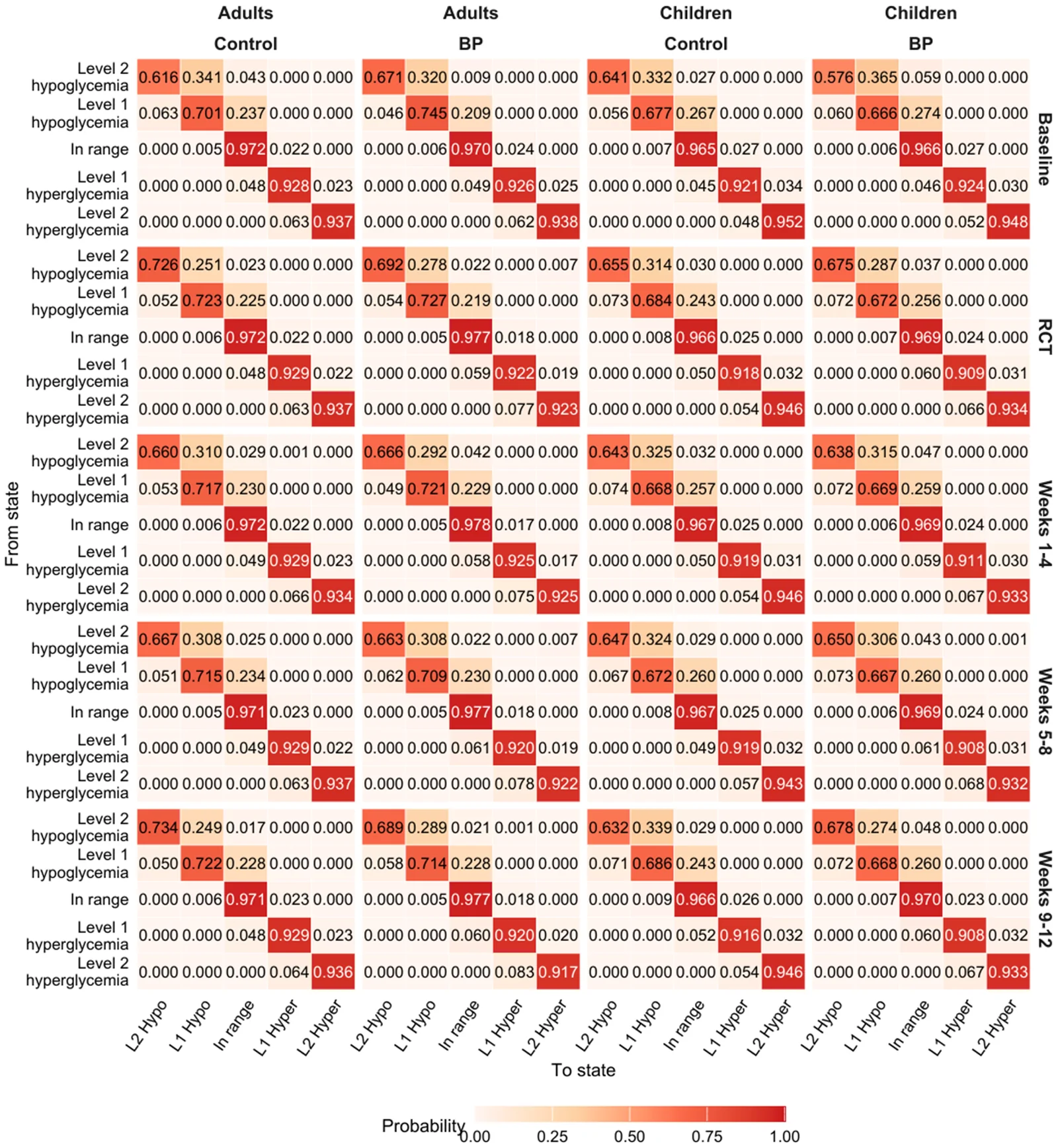
Heatmaps display the mean empirical transition probabilities between glycemic states for each group, stratified by age (adults, children), treatment (BP, Control), and study period (Baseline, RCT, Weeks 1–4, 5–8, 9–12). Each cell shows the average probability of transitioning from the glycemic state on the y-axis (“From”) to the state on the x-axis (“To”). Darker red indicates a higher probability. Row probabilities sum to 1 in the underlying matrices, but displayed values may not sum exactly to 1 because of rounding to three decimal places.

At baseline, adults and children in the BP and Control groups had broadly similar transition matrices, although pediatric comparisons should be viewed in light of the baseline mean glucose imbalance described above. In this type 1 diabetes cohort, the majority of transitions remain in the same glycemic state, and when movement occurs it most often goes to adjacent states. Transitions originating in Level 2 or Level 1 hyperglycemia and in range are dominated by self-transitions, whereas transitions starting in hypoglycemic states are based on fewer observations and are therefore less precise. This pattern is expected given the short allowable time gap between consecutive values (approximately 5 minutes) and the fact that there are many more observations in hyperglycemic and in-range states than in hypoglycemic states. It also aligns with the marginal probability plot (Fig 2), which shows a higher frequency of in-range and hyperglycemic states than hypoglycemic states.

As the trial progresses, the transition patterns between the BP and Control arms begin to diverge. During the full RCT and across the RCT sub-periods, adults and children receiving BP show slightly higher probabilities of moving from hyperglycemic states back into the in-range state compared with Control. The BP arm also shows higher probabilities of transitions from Level 2 to Level 1 hyperglycemia and longer dwell time in range. Although the absolute differences in transition probabilities are modest, they describe a clinically interpretable pattern of more frequent recovery from hyperglycemia and are consistent with the marginal probability distributions.

### Within-Participant glycemic transition dynamics

To investigate the effect of BP therapy on glycemic patterns within individuals, we examined changes in state-to-state glucose transition probabilities from the Baseline period to the RCT. Heatmaps in Fig 4 show the mean change in transition probabilities between glycemic states from baseline to RCT (RCT minus baseline). The exact Baseline and RCT probabilities underlying these changes are provided in S1 Fig. Among participants assigned to BP, we observed a higher probability of transitions into the in-range state from nearly all starting states.

**Fig 4:**
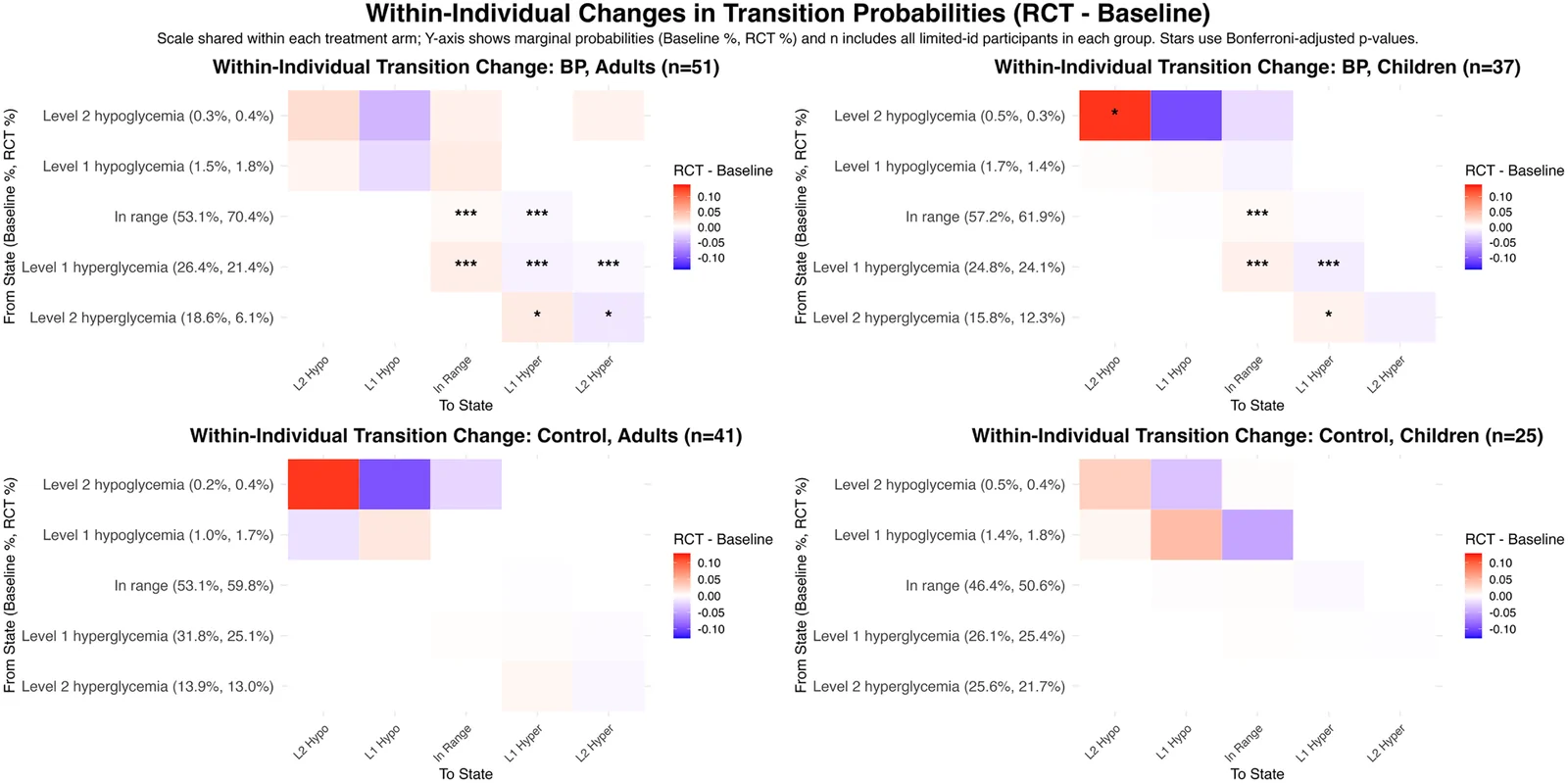
Heatmaps show the mean change in transition probabilities between glycemic states from baseline to the randomized trial period (trial minus baseline): positive (red) for increases and negative (blue) for decreases, stratified by treatment (BP, Control) and age group (adults, children). Y-axis labels give the marginal probability for each state in baseline and RCT periods. Statistical significance is based on bootstrapped p-values (* p < 0.05, ** p < 0.01, *** p < 0.001).

For adults on BP therapy with sufficient CGM data for analysis at both Baseline and RCT, within-person recovery from hyperglycemia increased from Baseline to RCT. In adults, Level 2 to Level 1 hyperglycemia rose from 0.061 to 0.075 (+0.014) and Level 1 hyperglycemia to in-range rose from 0.047 to 0.060 (+0.013). In children, Level 2 to Level 1 hyperglycemia increased from 0.058 to 0.068 (+0.010) and Level 1 hyperglycemia to in-range increased from 0.047 to 0.057 (+0.010). Bootstrap inference with Bonferroni correction supported statistically significant shifts toward in-range with BP, with confidence intervals shown in S2 Fig.

Within-person changes in the Control arm were minimal and not significant after Bonferroni correction. In adults, Level 2 to Level 1 hyperglycemia increased by +0.005 and Level 1 hyperglycemia to in-range by +0.001. In children, the corresponding increases were +0.001 and +0.002. This pattern is consistent with more frequent recovery from hyperglycemia during BP therapy, whereas Control group participants showed only modest changes. In the adult Control group, some hypoglycemia transition cells showed relatively large estimated changes, such as Level 2 hypoglycemia to Level 1 hypoglycemia, but these estimates were based on rare states and did not reach statistical significance after Bonferroni correction. Because Level 1 and Level 2 hypoglycemia were uncommon, estimates involving these rows can be influenced by a small number of observed transitions and should be interpreted cautiously.

### Between-Group glycemic transition dynamics

We also evaluated differences in glycemic transition dynamics between BP and Control groups across baseline, the overall RCT, and RCT sub-periods (Fig 5). Across RCT periods, we observed differences in how participants moved between glucose states with BP compared with Control. At baseline, adults in the BP and Control groups had similar transition patterns. Pediatric baseline comparisons should be interpreted cautiously because of the baseline mean glucose imbalance described above. Similar to the adult results, estimates for transitions out of hypoglycemia in children also require caution because Level 1 and Level 2 hypoglycemia states were uncommon.

**Fig 5:**
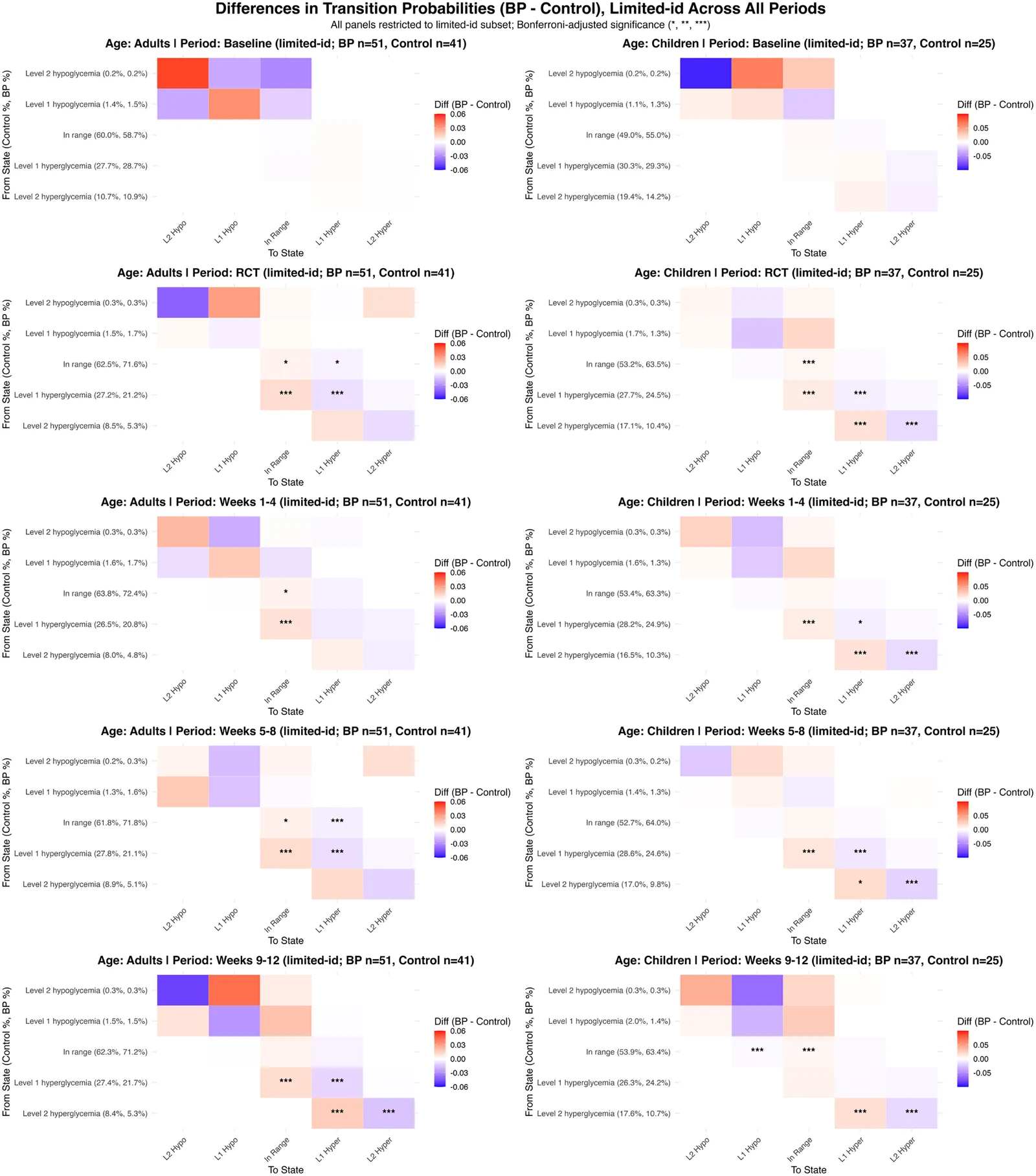
Heatmaps display the difference in transition probabilities between the BP and Control groups (BP minus Control) across all five glycemic states, stratified by age group and study period (Baseline, RCT [weeks 1–12], and RCT Sub-periods: weeks 1–4, 5–8, and 9–12). Red shades indicate transitions more frequent in the BP group; blue shades represent those more frequent in the Control group. Statistical significance is based on bootstrapped p-values (* p < 0.05, ** p < 0.01, *** p < 0.001).

Once the RCT began, BP treatment was associated with consistent changes in transition probabilities. Both adults and children with BP showed larger increases in transitions from hyperglycemia to in-range states. Many of these changes were statistically significant after bootstrap inference with Bonferroni correction. The corresponding bootstrap-based confidence intervals for these between-group differences are shown in S3 Fig.

In adults during the RCT, BP increased recovery from hyperglycemia versus Control: Level 1 hyperglycemia to in-range was 0.059 vs 0.048 (+0.011), and Level 2 to Level 1 hyperglycemia was 0.077 vs 0.063 (+0.014). These differences were consistent across RCT Sub-periods.

Children showed the same direction with smaller effects: During the full RCT period, Level 1 hyperglycemia to in-range was 0.060 vs 0.050 (+0.010) and Level 2 to Level 1 hyperglycemia was 0.066 vs 0.054 (+0.012). The largest Sub-period differences were +0.012 (Level 1 hyperglycemia to in-range, weeks 5–8) and +0.013 (Level 2 to Level 1 hyperglycemia, weeks 9–12).

A sensitivity analysis applying stricter data inclusion criteria, matching the within-participant analytic subset, showed similar patterns of between-group differences across time periods (S4 Fig).

### Comparison with traditional glycemic metrics

To place the empirical transition probability results in context, we compared them with conventional measures of glycemic variability and control, including MODD, MAGE, GRI, mean glucose, SD, CV, and percent time-in-range (TIR). Density plots illustrate the distributional differences in these metrics by treatment group and age (S5 Fig). Given non-normality in some distributions, the Wilcoxon rank-sum test was used for between-group comparisons (BP vs Control). These analyses were intended to provide descriptive context rather than reproduce the baseline-adjusted primary trial models.

Adults assigned to BP achieved significant improvements according to several conventional CGM metrics, including lower mean glucose, SD, and GRI and higher percent TIR compared with Control (Fig 6). MAGE differed significantly between BP and Control in adults, although its computation required more processing time than other metrics. MODD and CV were not statistically significant in adults. For children, BP therapy resulted in significantly lower mean glucose and GRI and higher TIR, but no significant differences were observed for MAGE, MODD, or CV. These findings suggest that transition probability matrices summarize a different aspect of CGM dynamics than several commonly reported variability metrics.

**Fig 6:**
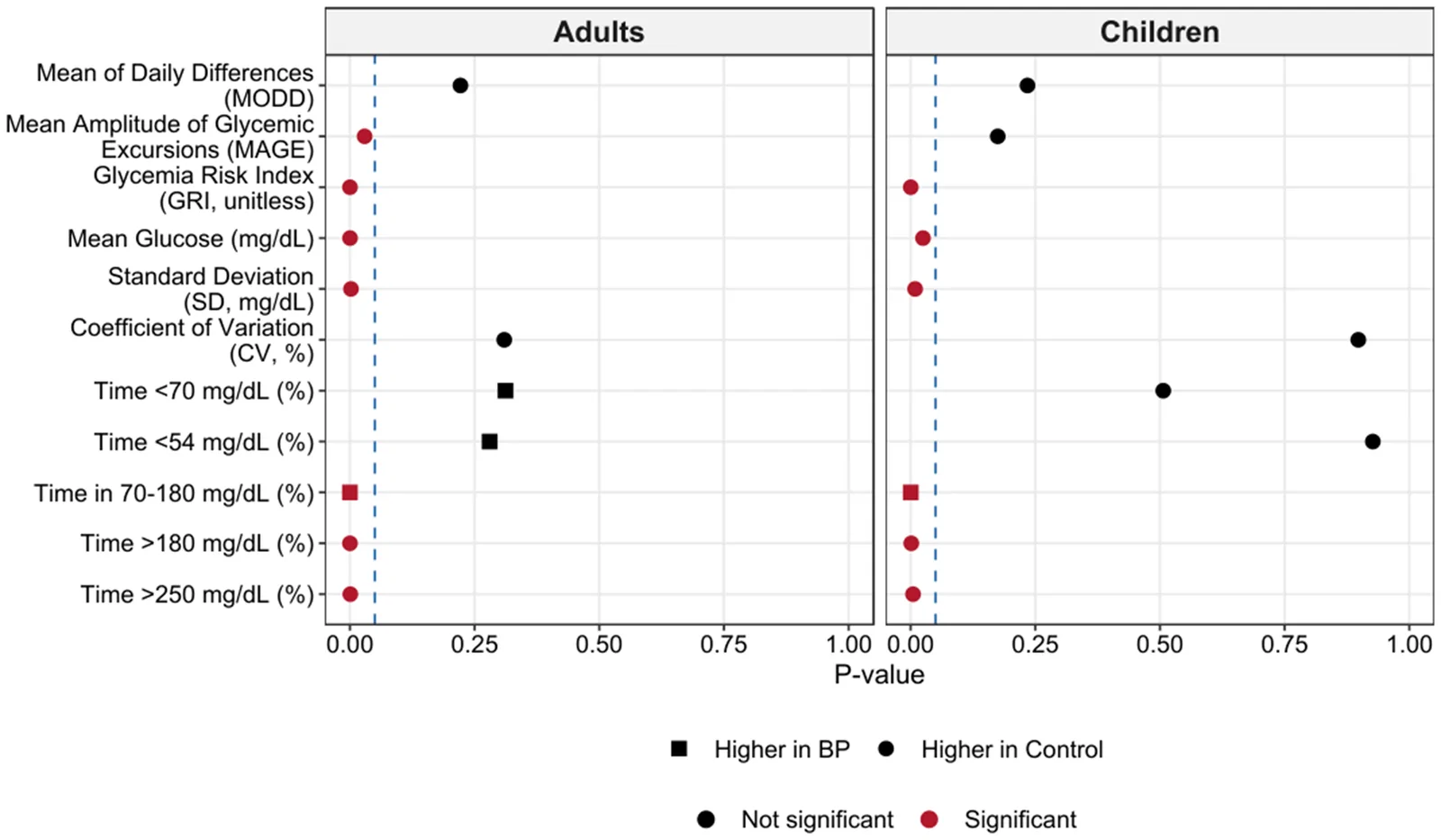
Forest plot displaying p-values from Wilcoxon rank-sum tests for between-group differences in glycemic metrics comparing BP and Control groups, stratified by adults and children. Squares indicate metrics with higher median values in BP; circles indicate metrics higher in Control. Red symbols denote statistically significant differences (p < 0.05), black symbols indicate non-significant differences. The vertical dashed line marks the p = 0.05 threshold.

## Discussion

The aim of this study was to evaluate whether empirical transition probability matrices can summarize sequential CGM patterns in a way that complements established CGM metrics. We found that transition matrices captured treatment-associated differences in movement from hyperglycemia toward the in-range state in the IOBP2 dataset. Existing CGM metrics, such as mean glucose and TIR, summarize the overall level and distribution of glucose values over time but do not directly account for the sequence or direction of glucose changes. Dynamic metrics such as MODD and MAGE describe aspects of temporal glucose dynamics, but their between-group patterns in this analysis differed from those observed in the transition matrices.

The transition probability approach explicitly captures how glucose moves between clinically defined states from one timepoint to the next. The visual format may be useful for clinicians and people living with diabetes and is consistent with the intuitive presentation of ambulatory glucose profile reports used in clinical care [29]. For example, it shows the empirical likelihood of returning to the in-range state after hyperglycemia or hypoglycemia. The approach is largely empirical, requires few modeling assumptions, and can be viewed as a strong complement to standard CGM summaries.

In the pediatric cohort, transition probability analysis reflected treatment-associated differences in hyperglycemia recovery even though several conventional variability metrics did not show statistically significant BP-Control differences in our unadjusted comparisons. This finding suggests that transition probabilities may provide additional descriptive information about glucose movement, particularly when the question concerns recovery from hyperglycemia or persistence within a glycemic state. Because the pediatric treatment groups differed in baseline mean glucose, these unadjusted comparisons should be interpreted cautiously.

Despite these strengths, the transition probability method has limitations. It discretizes continuous glucose into fixed categories, such as 70–180 mg/dL for ‘in range’, which can introduce boundary sensitivity: small fluctuations or measurement error near cut points may bias estimated transitions. Relatedly, a tighter range (70–140 mg/dL) is unlikely to change our conclusions because it is strongly correlated with 70–180 mg/dL time-in-range and would mainly reclassify intermediate hyperglycemia [30,31]. The same discretization also yields less precise estimates for rare states, especially Level 2 hypoglycemia in the IOBP2 data, because few observations contribute to these transitions as some participants never enter the state during a given period. These estimates should be interpreted cautiously. Hidden Markov Models (HMMs) offer a possible way to address these problems. Unlike our current approach, HMMs can model uncertainty in state assignment and smooth transitions around boundaries [32]. They may also produce more stable estimates in rare states by borrowing information across the available data. Future work could extend our framework using HMMs to address these limitations.

Another limitation is missing CGM data. We excluded transitions with time gaps greater than 6 minutes or less than or equal to 4 minutes to reduce bias from nonconsecutive readings and device irregularities, but this exclusion also led to information loss. Recent work suggests that this level of missingness is unlikely to meaningfully bias summary CGM metrics if missingness is random [33]. Future work could combine transition modeling with methods that more directly handle missingness, such as models that integrate HMMs with imputation [34].

Several statistical and design limitations also affect interpretation. First, the 25 transition probability cells are correlated because rows of a transition matrix sum to one and adjacent glycemic states are biologically related. Although we used Bonferroni correction, this approach is conservative and does not fully address the dependence structure among cells. Future work could use multivariate, hierarchical, or permutation-based approaches that account for the matrix structure more directly [35–37]. Second, our comparisons with conventional CGM metrics used unadjusted Wilcoxon rank-sum tests, whereas the original IOBP2 trial used baseline-adjusted models, and the pediatric cohort had a baseline imbalance in mean glucose. Future work should incorporate baseline adjustment into between-group transition probability estimation. Finally, this study did not test whether transition probability summaries improve prediction of clinically meaningful outcomes such as future HbA1c, severe hypoglycemia, diabetes complications, or quality of life.

In summary, transition probability matrices provide a clear descriptive summary of glucose dynamics that complements established CGM metrics. In the IOBP2 dataset, they showed treatment-associated patterns of more frequent recovery from hyperglycemia with BP therapy. Future validation studies should evaluate whether transition-based summaries improve prediction of clinically meaningful outcomes and whether they add value beyond established CGM measures in baseline-adjusted analyses and clinical decision-making.

## Supporting information

S1 File Supplementary methods and tables. Contains Appendix A, defining marginal glycemic-state probabilities and conventional glycemic variability metrics, and Supplementary Tables 1, analytic sample sizes by age group, treatment group, and study period after application of CGM completeness criteria, and S2 Table, number of participants who did not enter selected glycemic states, by analysis type, treatment group, and age group.
(DOCX)

S1 Fig Mean empirical transition probability matrices by age group, treatment group (BP, Control), and study period (Baseline, RCT). Rows represent “From” states and columns represent “To” states; darker red indicates higher transition probabilities.
(TIFF)

S2 Fig Mean within-participant changes in transition probabilities from Baseline to RCT (RCT minus Baseline), stratified by treatment and age group. Cells show mean changes with 95% bootstrap confidence intervals; red indicates increases and blue indicates decreases.
(TIFF)

S3 Fig Differences in transition probabilities between BP and Control (BP minus Control), stratified by age group and study period. Cells show mean differences with 95% bootstrap confidence intervals; red indicates higher probabilities in BP and blue indicates higher probabilities in Control.
(TIFF)

S4 Fig Sensitivity analysis of BP-Control differences in transition probabilities among participants meeting completeness criteria during both Baseline and all RCT Sub-periods. Red indicates higher probabilities in BP and blue indicates higher probabilities in Control; significance is based on bootstrapped p-values.
(TIFF)

S5 Fig Distributions of conventional CGM metrics by treatment group and age group. Shapiro–Wilk test p-values for normality are shown within each panel.
(TIFF)
