# Supplementary material for: Empirical transition probability modeling of continuous glucose monitoring data
Source: PLoS One. 2026 Oct 6;21(10):e0358361. doi: 10.1371/journal.pone.0358361 (PMC13641706; doi:10.1371/journal.pone.0358361)
Supplement: Supplementary file 1 [file pone.0358361.s001.docx]

Transition Probability Modeling of Continuous Glucose Monitoring Data

Supplementary Material

Yuanzhi Yu MS^1^, Margaret Banker PhD^1^, Jennifer Sherr MD, PhD^2^, Denise M Scholtens PhD^1^

**Author Affiliations:** ^1^Northwestern University Feinberg School of Medicine, Department of Preventive Medicine, Division of Biostatistics and Informatics, Chicago, IL 60611

^2^Department of Pediatrics, Yale School of Medicine, New Haven, CT 06511

**Corresponding author:** Denise M Scholtens, Northwestern University Feinberg School of Medicine, Department of Preventive Medicine, Division of Biostatistics and Informatics, 680 N Lake Shore Drive, Suite 1400, Chicago, IL 60611; dscholtens@northwestern.edu.

Yuanzhi Yu, MS

Northwestern University Feinberg School of Medicine

Department of Preventive Medicine, Division of Biostatistics and Informatics

680 N Lake Shore Drive Suite 1400

Chicago, IL 60611

[yuanzhi.yu@northwestern.edu](mailto:yuanzhi.yu@northwestern.edu)

Margaret Banker, PhD

Northwestern University Feinberg School of Medicine

Department of Preventive Medicine, Division of Biostatistics and Informatics

680 N Lake Shore Drive Suite 1400

Chicago, IL 60611

[margaret.banker@northwestern.edu](mailto:margaret.banker@northwestern.edu)

Jennifer Sherr, MD, PhD

Yale School of Medicine

Department of Pediatrics

One Long Wharf Drive Suite 503

New Haven, CT 06520

[jennifer.sherr@yale.edu](mailto:jennifer.sherr@yale.edu)

Denise M Scholtens, PhD

Northwestern University Feinberg School of Medicine

Department of Preventive Medicine, Division of Biostatistics and Informatics

680 N Lake Shore Drive Suite 1400

Chicago, IL 60611

312-503-7261

[dscholtens@northwestern.edu](mailto:dscholtens@northwestern.edu)

**Appendix A**

**Marginal Probability**:

For each participant, $u=1,\ldots,N_{\text{group}}$, and glycemic state $j=1,\ldots,S$, the marginal probability $\pi_{j}^{\left( u \right)}$ is defined as:

$$\pi_{j}^{\left( u \right)}=\frac{n_{j}^{\left( u \right)}}{n_{\text{total}}^{\left( u \right)}}$$

where $n_{j}^{\left( u \right)}$ is the number of CGM measurements assigned to state $j$ for participant $u$, $n_{\text{total}}^{\left( u \right)}$ is the total number of observed CGM measurements for participant $u$ during the period.

To obtain group-level summaries, we averaged these individual marginal probabilities across all $N_{\text{group}}$ participants within each analysis group (defined by treatment arm, age group, and study period as described above):

$$\pi_{j}^{\text{group}}=\frac{1}{N_{\text{group}}}\sum_{u=1}^{N_{\text{group}}} \pi_{j}^{\left( u \right)}$$

**Glycemia Risk Index** (GRI)[1]:

$$GRI = 3.0\cdot\left( P_{L2Hypo}+ 0.8\cdot P_{L1Hypo} \right)+ 1.6\cdot\left( P_{L2Hyper}+ 0.5\cdot P_{L1Hyper} \right)$$

where $P_{L2Hypo}$, $P_{L1Hypo}$, $P_{L2Hyper}$, $P_{L1Hyper}$are the percent time of CGM values in Level 2 hypoglycemia, Level 1 hypoglycemia, Level 1 hyperglycemia and Level 2 hyperglycemia.

**Mean of Daily Differences** (MODD)[2]:

$$MODD=\frac{1}{D-1}\sum_{d=1}^{D-1} \frac{1}{n_{d}}\sum_{t=1}^{n_{d}} \left| G_{d+1}\left( t \right)-G_{d}\left( t \right) \right|,$$

where $G_{d}\left( t \right)$ is glucose at time $t$ on day $d$, $D$ is the total number of days, and $n_{d}$ is the number of matched time points between day $d$ and day $d+1$.

**Mean Amplitude of Glycemic Excursions** (MAGE)[3]:

Computed as the mean amplitude of significant intraday glucose excursions exceeding daily standard deviation:

$$MAGE=\frac{1}{m}\sum_{k=1}^{m} E_{k},$$

where $E_{k}$ is the amplitude, the difference between the peak and nadir within a day, of the $k^{\text{th}}$ significant excursion and $m$ is the number of such excursions. Significant excursion is defined as any intraday glucose swing that exceeds the standard deviation of glucose values for that day[3].

**Supplemental Table 1:** Analytic sample sizes by age group and treatment arm after applying CGM completeness criteria. RCT analyses required ≥80% CGM coverage in each RCT sub-period (weeks 1–4, 5–8, and 9–12). Baseline analyses required at least 14 days of CGM data before randomization (days need not be consecutive), consistent with the primary trial criteria. Within-person analyses required participants to meet both the Baseline and RCT completeness rules.

| **Analysis set** | **Adults: Total** | **Adults: BP** | **Adults: Control** | **Children: Total** | **Children: BP** | **Children: Control** | **Overall Total** |
| --- | --- | --- | --- | --- | --- | --- | --- |
| Baseline analyses | 118 | 73 | 45 | 85 | 52 | 33 | 203 |
| RCT analyses | 121 | 72 | 49 | 123 | 78 | 45 | 244 |
| Within-person analyses | 92 | 51 | 41 | 62 | 37 | 25 | 154 |

**Supplemental Table 2:** Number of participants who never entered specific glycemic states at Baseline or during the RCT, by analysis type, treatment group, and age. Counts are based on participants included in each respective analytic subset.

| **Analysis Context** | **Age Group** | **Treatment Group** | **Level 2 Hypoglycemia** | **Level 1 Hypoglycemia** | **Level 2 Hyperglycemia** |
| --- | --- | --- | --- | --- | --- |
| **Between-Group (Baseline)** | Adults | BP | 14 | 3 | 0 |
| **Between-Group (Baseline)** | Adults | Control | 7 | 2 | 1 |
| **Between-Group (Baseline)** | Children | BP | 11 | 2 | 0 |
| **Between-Group (Baseline)** | Children | Control | 7 | 2 | 1 |
| **Between-Group (RCT)** | Adults | Control | 1 | 0 | 0 |
| **Within-Group (Baseline)** | Adults | BP | 9 | 1 | 0 |
| **Within-Group (Baseline)** | Adults | Control | 5 | 2 | 1 |
| **Within-Group (Baseline)** | Children | BP | 5 | 1 | 0 |
| **Within-Group (Baseline)** | Children | Control | 6 | 2 | 1 |
| **Within-Group (RCT)** | Adults | Control | 1 | 0 | 0 |

**Reference**

1. Klonoff DC, Wang J, Rodbard D, et al. A Glycemia Risk Index (GRI) of Hypoglycemia and Hyperglycemia for Continuous Glucose Monitoring Validated by Clinician Ratings. J Diabetes Sci Technol 2023;**17**(5):1226-42 doi: 10.1177/19322968221085273 [published Online First: 20220329].

2. Molnar GD, Taylor WF, Ho MM. Day-to-day variation of continuously monitored glycaemia: a further measure of diabetic instability. Diabetologia 1972;**8**(5):342-48.

3. Service FJ, Molnar GD, Rosevear JW, Ackerman E, Gatewood LC, Taylor WF. Mean amplitude of glycemic excursions, a measure of diabetic instability. Diabetes 1970;**19**(9):644-55 doi: 10.2337/diab.19.9.644.
